# Gamma Knife Radiosurgery: An Adjuvant Therapy for Primary Sellar Paraganglioma

**DOI:** 10.7759/cureus.56228

**Published:** 2024-03-15

**Authors:** Madan Bajagain, Shingo Fujio, Mari Kirishima, Kazutaka Yatsushiro, Ryosuke Hanaya

**Affiliations:** 1 Department of Neurosurgery, Graduate School of Medical and Dental Sciences, Kagoshima University, Kagoshima, JPN; 2 Department of Pathology, Graduate School of Medical and Dental Sciences, Kagoshima University, Kagoshima, JPN; 3 Department of Neurosurgery, Fujimoto General Hospital, Miyakonojo, JPN

**Keywords:** transsphenoidal surgery, transcranial surgery, primary sellar paraganglioma, gamma knife stereotactic radiosurgery, adjuvant therapy

## Abstract

Sellar paraganglioma (SP) is a rare benign tumor, usually treated by surgery. SPs are lobulated, firm, adherent, and highly vascular, allowing mostly partial resection. We present the case of a 52-year-old man diagnosed with primary SP, treated with a transcranial-transsphenoidal (TC-TS) surgical approach, followed by adjuvant Gamma Knife stereotactic radiosurgery (GKSR). The tumor has an extra-pituitary origin, with a sellar-suprasellar, right cavernous sinus extension that encroached the bilateral optic nerve and anterior cerebral artery. Histopathology confirmed SP with a Zellballen pattern. Despite postoperative tumor growth observed at four and 10 months, a stable residual tumor was noted at a follow-up two years after GKSR. SP is diagnosed mainly in middle age or in adolescent males. The TC-TS approach offers a bidirectional view that allows greater resection by minimizing blind spots, thus reducing complications. Similar to the paragangliomas of other sites, the efficacy of GKSR was observed for primary SP. SP is a rare differential diagnosis of pituitary diseases; however, it should be considered. After surgical resection of primary SP, GKSR is observed as an effective adjuvant therapy.

## Introduction

Primary sellar paraganglioma (SP) is a rare benign pituitary disease derived from neural crest progenitor cells [1]. The usual modality of treatment for SP is surgical resection using a transcranial (TC) or transsphenoidal (TS) approach. However, in most previous cases, only partial resections or biopsies were achieved due to the firm consistency, adherence, and high vascularity of the tumor [2,3]. SP may also exhibit recurrence and usually requires adjuvant radiation therapy. Here, we present a case of a primary SP treated with adjuvant Gamma Knife stereotactic radiosurgery (GKSR) after a TC-TS surgery.

This article was previously presented as a meeting abstract at the 32nd Annual Meeting of the Japanese Society for Hypothalamic and Pituitary Tumors on February 18, 2022.

## Case presentation

**Figure 1:**
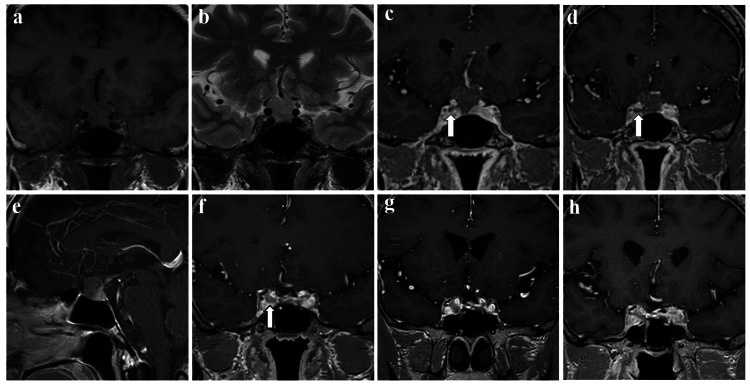
Gradual changes on magnetic resonance imaging (a, b) At the initial visit, coronal images showed a solid tumor extending into the suprasellar region (a: T1-weighted, b: T2-weighted); (c, d, e) T1 contrast images showed an extra-pituitary origin of the tumor with the normal pituitary gland and the stalk situated caudally to the left (c, d: coronal, e: sagittal). The contrast effect of the tumor was weak. Tumor invasion into the right cavernous sinus was suspected (arrow in c and d); (f, g) Postoperative T1 contrast coronal images revealed a residual tumor in the right cavernous sinus (arrow in f), which increased in size (f: four months: maximum tumor size was 4 mm, g: 10 months: maximum tumor size was 7 mm); (h) Coronal T1 contrast images taken two years after Gamma Knife stereotactic radiosurgery showed no further tumor growth (The maximum tumor size was 6 mm).

A 52-year-old man with occasional headaches underwent head computed tomography (CT) that revealed a sellar mass. Magnetic resonance imaging (MRI), performed following routine pituitary MRI protocol [4], showed a solid tumor extending into the sellar-suprasellar region with an isointense signal on T1- and T2-weighted imaging (Figure 1a, 1b). The tumor encroached on the A1 segment of the anterior cerebral artery on both sides, compressing the bilateral optic nerves. T1 contrast imaging suggested that the tumor had an extra-pituitary origin, with a normal pituitary gland located laterally and caudally (Figure 1c-1e). The contrast effect of the tumor was weak, and the dynamic study also showed minimal tumor contrast. Tumor invasion into the right cavernous sinus was suspected (Figure 1c, 1d).

The basal pituitary hormonal profile was normal. Further, an ophthalmological examination revealed no visual field defects. The first differential diagnosis was an ectopic pituitary adenoma. However, the tumor was less contrasted, which is atypical in pituitary adenomas. Rare tumors were also considered as differentials. Due to concerns about unusual postoperative bleeding and adhesion between the optic nerve and tumor, a combined TC and endoscopic TS surgery was performed.

**Figure 2:**
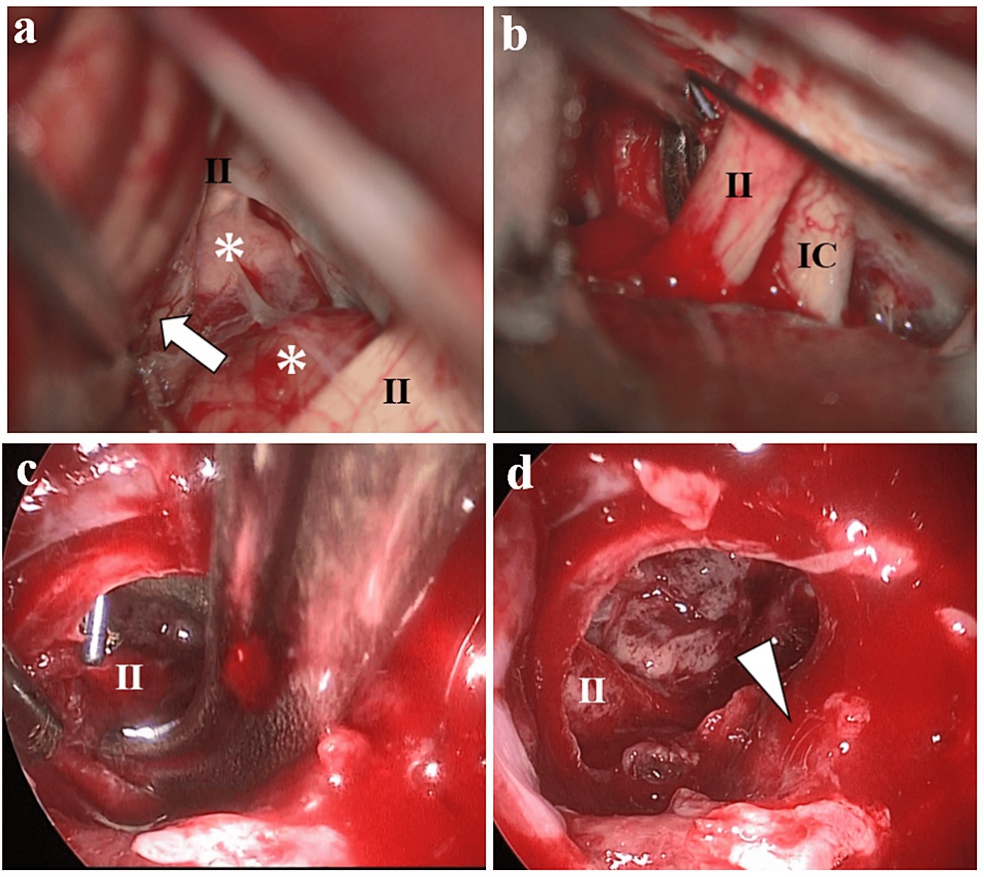
Intraoperative views of the transcranial side (a, b) and transsphenoidal side (c, d) (a) The tumor was located between the optic chiasm and attached to the left and right optic nerves (asterisk) and rectal gyrus (arrow); (b, c) The tumor under the right optic nerve was dissected via the transcranial approach and removed via the transsphenoidal route; (d) Final view. No tumor invasion into the pituitary gland was observed (arrowhead). II: optic nerve, IC: internal carotid artery.

The TC surgeon was positioned at the head of the operating table, whereas the TS surgeon was on the right side. The patient’s head was fixed in a Mayfield clamp and tilted 15° towards the left in a supine position. A high-definition endoscope (Karl Storz SE & Co. KG, Tuttlingen, Germany) was used to perform standard endoscopic endonasal TS surgery. After dissecting the bilateral nasal mucosa, the septal bone was harvested for sellar plasty. Next, the sella and dura were opened with a cruciate incision. The tumor center was soft, similar to a pituitary adenoma; however, the margins were fibrous and firm. The tumor border with the pituitary gland was easily detached; nonetheless, adhesion with the bilateral optic nerve, A1 segment of the anterior cerebral artery, and rectal gyrus was observed. The TC surgeon used the right pterional approach to dissect the tumor off the optic nerve, A1 segment of the anterior cerebral artery, and rectal gyrus, facilitating safer tumor resection and removal (Figure 2). Tumor invasion into the cavernous sinus was evident and excised as much as possible by the TS surgeon. The perioperative tumor bleeding was similar to that of a usual pituitary adenoma surgery. Adequate tumor removal with intact cavernous sinus was achieved.

**Figure 3:**
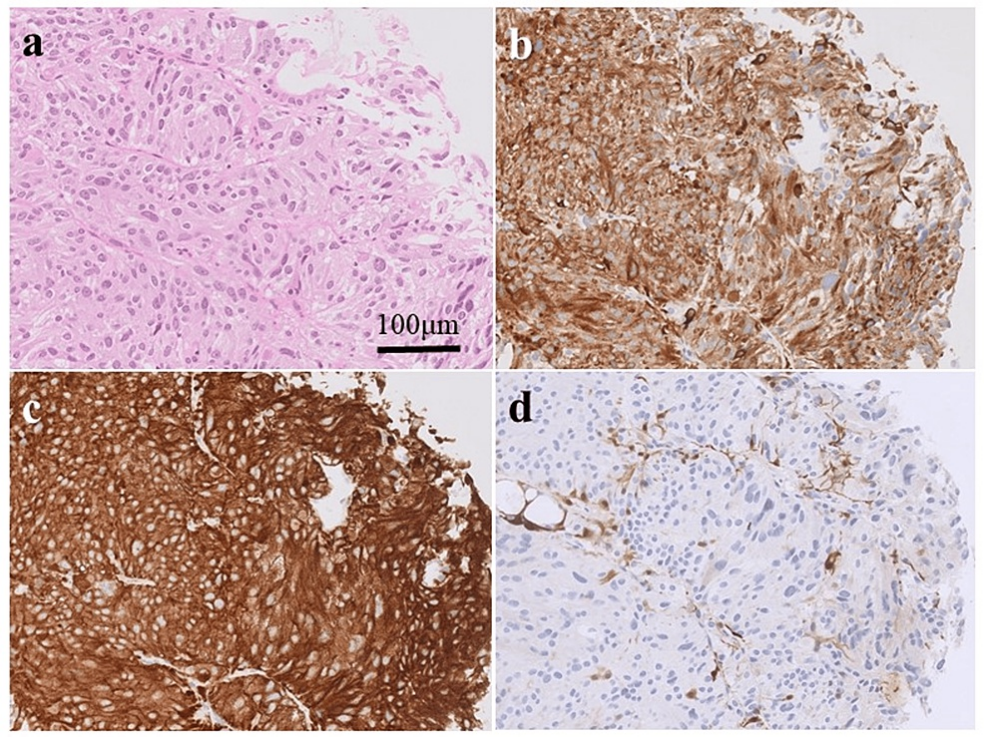
Pathological findings (a) Hematoxylin-eosin staining shows cytoplasmic tumor cells with a Zellballen appearance with large polymorphic nuclei and multinucleated cells; (b–d) Immunostaining showing tumor cells positive for proteins (b: chromogranin A, c: synaptophysin, d: S-100).

Histopathological examination revealed tumor cells with abundant cytoplasm, arranged in a nested Zellballen pattern (Figure 3a). Tumor cells were immunopositive for chromogranin A (Figure 3b) and synaptophysin (Figure 3c), whereas sustentacular cells surrounding the tumor were positive for S100 proteins (Figure 3d) and glial fibrillary acidic protein. Most tumor cells were negative for cytokeratin AE1/AE3 and anti-cytokeratin antibody 5.2, epithelial membrane antigen, vimentin, thyroid transcription factor-1, cluster of differentiation34, and signal transducer and activation of transcription6. The MIB-1 index was 2%; therefore, a diagnosis of SP was made. 

Whole-body CT and meta-iodobenzylguanidine scintigraphy revealed no detectable lesions with normal levels of urine metanephrine and normetanephrine. Corticotropin-releasing hormone, luteinizing hormone-releasing hormone, and thyrotropin-releasing hormone tests were performed three months after surgery; nevertheless, no decrease in anterior pituitary function was observed. Postoperative MRI at four months showed a residual tumor confined in the right cavernous sinus (Figure 1f), which increased in size at 10 months (Figure 1g).

GKSR with a marginal dose of 15 Gy was chosen over a second surgery as tumor regrowth was limited to the cavernous sinus. Two years after GKSR, no additional tumor growth was observed (Figure 1h). The pituitary function was also preserved.

## Discussion

Paragangliomas are benign neuroendocrine tumors that arise from paraganglionic tissue, constituting only 0.6% of all head and neck region tumors [5]. They are primarily located in the carotid body (60% to 70%), the jugular glomus, and along the vagus nerve [2]. SPs are relatively rare due to the absence of paraganglia in the sellar region, their origin and disease causation remain unknown. Meta-iodobenzylguanidine scintigraphy is a useful diagnostic tool for neuroendocrine tumors like paragangliomas [6]. However, this case exhibited no accumulation other than that in the pituitary gland, and the disease appeared to be primary.

A literature review on sellar region paraganglioma (excluding metastasis) yielded 30 reports, comprising 32 patients (Table 1).

**Table 1:** Literature review on sellar (region) paraganglioma. CS, cavernous sinus; CRT, conventional radiation therapy; c, cycle; F, female; f, fraction; fSRT, fractionated stereotactic radiation therapy; GKSR, Gamma Knife stereotactic radiosurgery; Gy, Gray; GTR, gross total resection; Lt., left; M, male; m, months; ND, no data; PS, parasellar; RT, radiation therapy; Rt., right; SPS, sphenoid sinus; STR, subtotal resection; SS, suprasellar; TC, transcranial route; TS, transsphenoidal route; *-once, **-twice, ***-thrice.

S.N.	First author and year of publication	Age/ Sex	Location	Surgical route/ Approach	STR/ GTR	Radiation therapy/ doses	
1	Chytil, 1967 [7]	46/M	Sellar / SPS	TS	STR	RT	CRT 40 Gy**
2	Bilbao et al., 1978 [8]	37/M	Sellar	TC	GTR	No RT	-
3	Ho et al., 1982 [9]	65/M	CS / semilunar ganglion	TC/ Rt. fronto temporal	STR	RT	CRT 52 Gy
4	Prabhakar et al. 1984 [10]	7/F	PS, pre and retro sellar	TC/ Rt. fronto temporal	STR	RT	CRT 45 Gy
5	Steel et al., 1993 [11]	44/F	Sellar, PS, and CS	TS	STR	RT	CRT 45 Gy
		41/F	Sellar, PS, and CS	TC/ Lt. fronto temporal	STR	RT	CRT 50 Gy
6	Flint et al., 1993 [12]	17/M	Sellar, SS, CS, and Carotid Sinus	TC/ sub frontal**	Biopsy, STR	No RT	-
7	Scheithauer et al., 1996 [1]	14/M	Sellar/ SS	TS, 3 m later TC/ Rt. frontal	Biopsy, STR	RT	CRT 50.4 Gy
8	Noble et al., 1997 [13]	71/M	Sellar	TC/ Rt. fronto- temporo-parietal	STR	ND	-
9	Mokry et al., 1998 [14]	76/M	Sellar / SS	TS, later TC/ Rt. pterional	STR	No RT	-
10	Del Basso De Caro et al., 1998 [15]	84/M	Sellar / SS	TS	GTR	No RT	-
11	Sambaziotis et al., 1999 [16]	54/M	Sellar	TS	GTR	No RT	-
12	Salame et al., 2001 [17]	48/F	Sellar / SS	TS	STR	No RT	-
13	Laquis et al., 2001 [18]	15/F	SS, CS	TC/ skull base	STR	RT	ND
14	Hertel et al., 2003 [19]	51/F	Sellar/ SS/ Middle cranial fossa and clival	TC/ Lt. trans-sylvian**	STR **	RT	fSRT
15	Arkha et al., 2003 [20]	58/F	Sellar/ PS	TC/ Lt. fronto- temporal	STR	No RT	-
16	Naggara et al., 2005 [21]	47/M	Sellar / SS	TC/ temporal	GTR	No RT	-
17	Zorlu et al., 2005 [22]	37/M	Sellar / PS	Multiple surgeries	GTR*** STR*	RT	CRT 50 Gy
18	Boari et al., 2006 [23]	52/M	Sellar	Sub-labial TS	STR	No RT	-
19	Voulgaris et al., 2006 [3]	48/M	Sellar / SS	TC/ fronto- temporal	STR	RT	ND
20	Peltier et al., 2007 [24]	51/F	PS	TC/ Lt. sub-frontal	STR	RT	CRT 45 Gy
21	Sinha et al., 2008 [25]	18/M	Sellar, SS and CS	TS	STR	No RT	-
22	Özüm et al., 2008 [26]	70/M	Sellar/ PS with B/L CS	TS	STR	RT	CRT 50 Gy
23	Haresh et al., 2009 [27]	17/M	Sellar/ PS	TS	STR	RT	fSRT 50 Gy 25f
24	Albert et al., 2011 [28]	63/M	Sellar/ PS with orbital extension	TC/ orbital osteotomy	STR, GTR	No RT	-
25	do Nascimento et al., 2012 [29]	33/F	Sellar	TS, 2 m later TC/ sub frontal	GTR	No RT	-
26	Li et al., 2017 [30]	40/F	Sellar / SS	TC/ sub-frontal	STR	RT	CRT 50 Gy
27	Karlekar et al., 2018 [31]	19/M	Sellar, PS with pontine extension	TC**	STR**	RT	ND
28	Lyne et al., 2019 [2]	73/F	Sellar / SS	TS	GTR	RT	ND
29	Vasoya et al., 2020 [32]	13/M	Sellar / SS	TC/ Left pterional	Biopsy	RT	CRT 50 Gy 25f
		20/M	Sellar / SS	TC/ Left pterional	Biopsy	RT	CRT 40 Gy 20f
30	Wang et al., 2023 [33]	70/F	Sellar / PS / SS	TC/ sub-frontal	STR	No RT	-
31	Current case	52/M	Sellar/ PS	Combined TS-TC / Rt. pterional	STR	RT	GKSR 15 Gy

Most patients with SP were diagnosed in middle age (40-59 years, 38.70%) or adolescence (10-19 years, 22.58%). SP is predominantly seen in males (65.63%), with a male-to-female ratio of 1.9:1, contrasting with the female-predominant carotid, vagal, tympanic, and laryngeal paragangliomas [34,35]. SP tumors are lobulated, firm, and highly vascular, with or without a capsule [1,33]. They are adherent to the carotid artery, cavernous sinus, and optic nerve, which extends to the extra sellar locations [2,3,33]. Although the exact origin of the SP is unknown, in the current case, the border between the tumor and the pituitary gland was clear, and the tumor extended into the cavernous sinus, suggesting that it may have originated from within the cavernous sinus. MRI of SP shows heterogeneous intensity on T2 with gadolinium enhancement, indicating high vascularity [12,32,33]. However, the findings were atypical for the present case, with a weak contrast effect and minimal enhancement in dynamic studies.

The primary treatment modality for SP is surgical resection via the TC or TS route. The preferred surgical approaches for TC were sub-frontal and trans-pterional. Preoperative endovascular embolization to devascularize the SP followed by TS resection is a plausible treatment option. In the late 20th-century case reports, SP patients were treated via both the TC [8-13] and TS [1, 7,11,14-16] routes equally (n=6 each). However, since 2000, more patients with SP have been treated via the TC route (13 patients, 65%) [3,18-22,24,28,30-33] rather than the TS route (seven patients, 35%) [2,17,23,25-27,29]. The primary treatment choices for SP are biopsy (12.5%), subtotal resection (65.6%), or gross total resection (21.9%). These facts show that reaching a consensus to follow a TC or TS route to achieve adequate tumor resection can be challenging. Recently, combined TC-TS surgeries have been employed for anterior skull-base tumors that include meningiomas, giant pituitary adenomas, and craniopharyngiomas [36]. The combined TC-TS approach allows bidirectional views for greater resection by minimizing blind spots and reducing residual tumor masses in the tumor bed. This prevents post-operative bleeding, particularly when the tumor extends beyond the capsule intracranially [36,37]. Surgical manipulation from two directions also facilitates precise dissection along the carotid artery and optic nerves [36,38]. The TS approach alone involves a high risk for postoperative hemorrhage, edema, and mass effects if radical resection is not achieved [37]. The tumor, in the current case, was without a capsule, encroached on the A1 segment of the anterior cerebral artery, and compressed the bilateral optic nerves, which guided our decision to pursue a combined TC-TS approach. Despite many previous reports achieving partial resections, we chose the combined technique for its potential to allow a gross safer resection despite the complex anatomical involvement.

A literature review on post-surgical adjuvant radiation therapy yielded 18 patients: four lacked relevant data [2,3,18,31], two received fractionated stereotactic radiation therapy [19,27], and the rest received conventional radiation therapy [1,7,9-11,22,24,26,30,32]. Most patients who underwent biopsy and subtotal resection (75% and 66.5%, respectively) received radiation therapy as an adjuvant. GKSR is effective for pituitary adenomas, metastatic tumors, meningiomas, schwannomas, and other benign tumors [39]. Additionally, GKSR is effective in treating paragangliomas found at other sites. A literature review of patients treated with GKSR for glomus jugulare tumors showed that 90.5% had tumor control [40]. Similarly, another study of 55 patients with a mean follow-up of 86.4 months had 94.8% of tumor control for jugulotympanic paragangliomas [41]. However, GKSR has neither been previously utilized nor reported in the literature for the treatment of SP recurrence. GKSR plays a crucial role in stabilizing tumor growth and preserving surrounding structures, especially in surgically inaccessible locations, such as the right cavernous sinus in this case. Follow-up MRI two years after the GKSR showed a stable tumor. This case provided an opportunity to investigate the effectiveness of GKSR in stabilizing SP recurrence. Further studies are warranted to elucidate the potential of GKSR as a novel therapeutic modality in this context.

## Conclusions

SP is a rare disease that should be considered in pituitary pathology, particularly in adolescent and middle-aged men. Complete surgical removal of a tumor is generally unattainable via the TC or TS approach alone. Hence, a combined simultaneous TC-TS surgical approach can enable a near-complete and safer resection. GKSR plays a crucial role in treating SP growth in surgically inaccessible locations.
